# Cas3 Protein—A Review of a Multi-Tasking Machine

**DOI:** 10.3390/genes11020208

**Published:** 2020-02-18

**Authors:** Liu He, Michael St. John James, Marin Radovcic, Ivana Ivancic-Bace, Edward L. Bolt

**Affiliations:** 1 School of Life Sciences, University of Nottingham, Nottingham NG7 2UH, UK; mbxlh7@nottingham.ac.uk (L.H.); mrs32@kent.ac.uk (M.S.J.J.); 2 Department of Biology, Faculty of Science, University of Zagreb, 10 000 Zagreb, Croatia; marin.radovcic@biol.pmf.hr

**Keywords:** CRISPR, Cas3, helicase, nuclease, biofilm

## Abstract

Cas3 has essential functions in CRISPR immunity but its other activities and roles, in vitro and in cells, are less widely known. We offer a concise review of the latest understanding and questions arising from studies of Cas3 mechanism during CRISPR immunity, and highlight recent attempts at using Cas3 for genetic editing. We then spotlight involvement of Cas3 in other aspects of cell biology, for which understanding is lacking—these focus on CRISPR systems as regulators of cellular processes in addition to defense against mobile genetic elements.

## 1. Introducing Cas3—The Identification of a DNA Helicase-Nuclease Machine 

**Figure 1:**
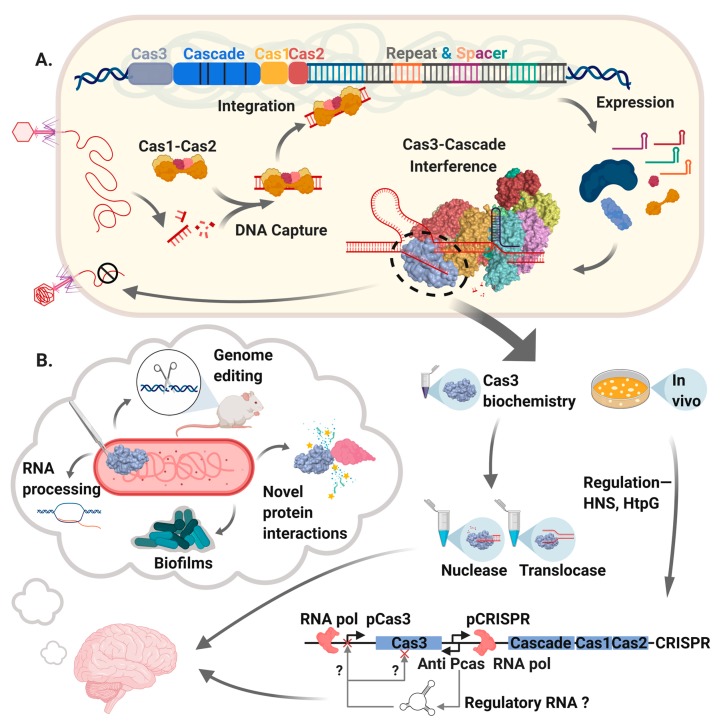
A summary of Cas3 as covered in this review. **A.** The main events in CRISPR-Cas systems that use Cas3. DNA from a mobile genetic element (bacteriophage shown here) is captured and integrated into a CRISPR locus by “Adaptation”, catalyzed by Cas1-Cas2 proteins helped by various non-Cas host proteins. Transcription of CRISPR generates RNA that after further processing is called CRISPR RNA (crRNA) that is loaded into a multi-protein effector complex, forming “Cascade”, which targets crRNA to the invader DNA. Cascade recruits Cas3 to targeted DNA forming the “Interference” complex that degrades DNA and in so doing can provide DNA for capture by Cas1-Cas2. We highlight Cas3 within the interference complex, leading into part **B.,** illustrating that studies of Cas3 enzymology in vitro have detailed Cas3 mechanism when associated with Cascade and in isolation. Genetic analyses of the *cas3* gene and its regulation in bacteria (e.g., by HtpG and H-NS) provoke ideas for additional roles of Cas3 in natural cellular physiology and in biotechnology. In the figure we highlight experimental observations that indicate potential roles for Cas3 in RNA processing and biofilm formation, and usefulness in genetic editing reactions.

In this article, we review original research that describes the structure and function of prokaryotic Cas3. Cas3 is an essential component of CRISPR-Cas adaptive immunity systems that repel invader genetic elements (reviewed recently [1,2,3]), but it also plays-in to several other aspects of cell biology, highlighted in Figure 1. Cas3 is an ATP-dependent single-strand DNA (ssDNA) translocase/helicase that in many CRISPR systems is fused to a HD-nuclease domain. The Cas3 translocase and nuclease activities degrade DNA by reducing it to shorter fragments of 10s of nucleotides, nullifying invader DNA as part of processes called ‘CRISPR interference’.

Cas3 structure and function is described in detail further on. Cas3 was first highlighted in 2002 during in silico analyses of prokaryotic genomes [4,5] that identified its superfamily-2 helicase motifs (as ‘COG1203’) [4], and its association with other proposed nucleic acid processing enzymes located alongside repeat DNA sequences [5].

This created the term ‘CRISPR’, for the DNA repeats, and the protein COG1203 became ‘Cas3’, a CRISPR-associated protein [5]—the discovery and establishment of CRISPR biology and biotechnology is described in [6]. A subsequent hypothesis proposed that Cas3 is part of an RNA-interference-based prokaryotic immunity system, ‘CRISPR-Cas’ [7], affirmed in a CRISPR system lacking Cas3 [8]—this particular system instead uses Cas9 for CRISPR interference reactions—but the importance of Cas3 was identified in *Escherichia coli*, functioning in CRISPR immunity with a ‘Cascade’ protein complex [9].

## 2. Cas3 in CRISPR Immunity—A Cut, Catch, and Reel Mechanism

Cas3 is a ‘signature’ protein of Type 1 CRISPR systems—classification and evolution of CRISPR systems is reviewed in [10,11,12,13]. In these systems Cas3 functions with a ribonucleoprotein complex called ‘Cascade’. Cascade base pairs CRISPR RNA (crRNA—one *per* Cascade) to a target DNA forming an R-loop [9,14,15,16,17]. Recruitment of Cas3 to a Cascade-R-loop therefore places Cas3 DNA nuclease-translocase activity in readiness for degradation of the target DNA. This is the overall basis of CRISPR interference in Type 1 CRISPR systems.

Diversity of Cascade-Cas3 form and function across Type 1 CRISPR systems is made explicit by the seven or eight subtypes; 1-A to F, 1-U and 1-F_variant_ [13]. Within these, Cascade complexes vary in composition from three to five protein subunits—although Cas5 and Cas7 are common to all subtypes—and show some corresponding variation in catalytic functions of the subunits during genesis of the crRNA payload and its targeting to DNA. Compare, for example, Cascade complexes from *Desulfovibrio vulgaris* (subtype 1-C) and *E. coli* (subtype 1-E) [14,18]. For Cas3 the most remarkable differences between subtypes are existence of Cas3 fusion and fission proteins. Fused Cas3 translocase-HD-nuclease proteins are fused further to the Cas2 adaptation enzyme in some *Yersinia* and *Pseudomonas* bacteria. In some archaea Cas3 functions are split into distinct HD-nuclease and translocase/helicase proteins that are encoded from different genes (respectively, Cas3’’ and Cas3’) [19,20] (and authors’ unpublished data). In some cyanobacteria Cas3’’ is fused to Cas10d protein [13]. We next assess Cas3 catalytic activities, focusing on the most common form as a fused HD-nuclease-translocase.

### 2.1. Cas3 Nuclease

**Figure 2:**
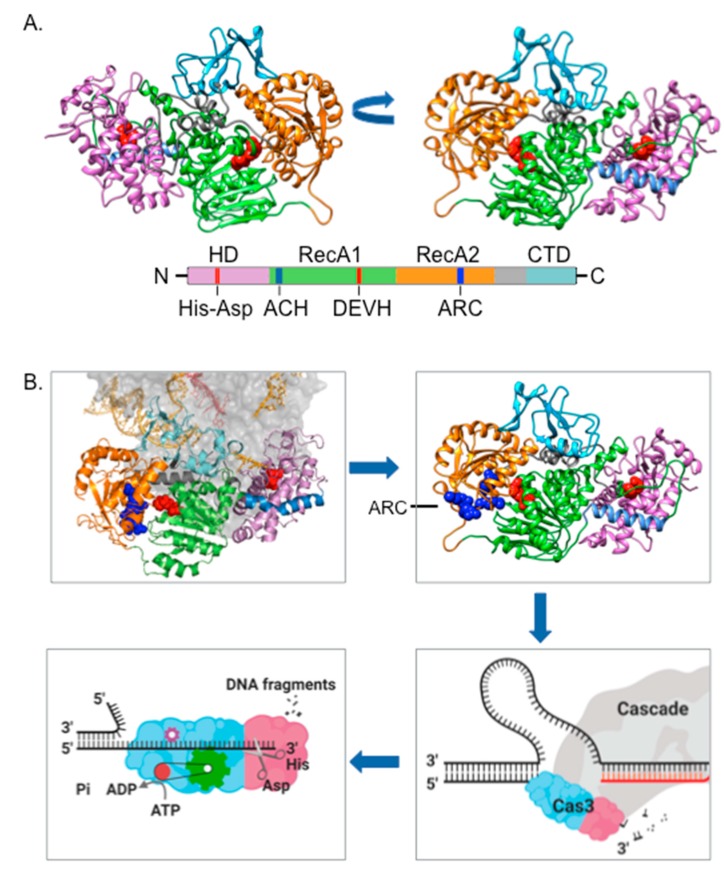
Cas3 structure-function. **A.** PHYRE2-predicted *Escherichia coli* Cas3 structure, modeled from Cas3 structures solved from *Thermobaculum terrenum* (PDB 4Q2C) and *Thermobifida fusca* (PDB 4QQW, 4QQX and 4QQY), represented in two orientations and with a corresponding cartoon of primary sequence presented below. We highlight the HD domain (purple), two RecA-like domains (green and orange) and the accessory C-terminal domain (CTD, pale blue). Active sites comprising the Asp-His HD domain and the amino acid DEVH motif of one RecA-like domain for ATP-hydrolysis are highlighted in red spheres within the structures, and marked on the cartoon primary structure. Also marked are the prominent solvent-exposed alpha helix (ACH) and an arginine rich channel (ARC) described in the main text. **B.** Panels should be followed from top left, clockwise. The location of Cas3 (using same domain colors as used in part A) bound to Cascade subunit protein Cse1 (grey), presented from the *T. fusca* Cascade-Cas3 structure (PDB: 6C66). DNA parts of the R-loop are colored orange—in CRISPR interference this DNA is nicked by the Cas3 HD domain, and then assimilated into the translocase/helicase active sites, possibly *via* interaction between DNA and the arginine residues of the ‘ARC’, highlighted as blue spheres. Captured single-strand DNA (ssDNA) is then translocated through the Cas3 protein by a reeling mechanism, which is associated with nuclease activity that generates DNA fragments.

Cas3 comprises a HD-nuclease domain fused to two RecA-like domains characteristic of superfamily-2 helicases (Figure 2). These co-operate to deliver ATP-dependent ssDNA translocation and degradation [21,22]. Several conserved features of Cas3 enzymes are likely to be important for co-ordination of ssDNA from RecA domains into the HD-active site, including a prominent solvent-exposed helix (labeled ACH in Figure 2A), and tyrosine/tryptophan residues located at the RecA-HD domain interface and elsewhere. In common with many helicases, Cas3 has an accessory domain positioned at the protein C-terminus, although its exact functions are not clear. HD-nuclease function can be supported by a variety of metal ions [19,22,23]. Crystal structures of Cas3 display HD-active sites bound to iron [24], manganese [25] and calcium (unpublished, PDB code—3M5F, 2011). Biochemistry of Cas3 in vitro indicates nuclease activity stimulated by manganese and cobalt, and inhibited by iron [23,26], and cobalt was necessary to observe stable interaction between Cas3 and Cascade in one system at least [27,28].

Critical to the effectiveness of Cas3 as a nuclease in CRISPR systems is that Cascade first validates a *bona fide* target DNA. Cascade identifies a DNA ‘PAM’ sequence (‘Protospacer Adjacent Motif’) at a target site, followed by stabilized DNA binding from RNA-DNA base-pairing, forming a Cascade R-loop that is ‘locked’ on to the target DNA [27,29,30,31]. In this state conformation of the Cse1 component of Cascade reveals Cas3 interacting regions. Cas3 will load onto the ssDNA that is generated in R-loops, but interaction with the Cse1 subunit of Cascade channels its nuclease activity for CRISPR immunity [18,31,32,33]. Analysis using Hydrogen-Deuterium Exchange (HDX) coupled to mass spectrometry identified alpha helix H1 of Cse1 as a critical docking and/or activation site for interaction with Cas3 [34], and may be the molecular trigger for degradation of targeted DNA by the Cas3 HD-nuclease activity [27,28].

Biochemical and structural analyses of Cas3 and Cas3-Cascade from *E. coli*, *Thermobaculum terrenum* and *Thermobifida fusca* have provided exciting evidence about their CRISPR interference mechanism. In particular, how target DNA is located by Cascade for R-loop formation, how this engages with Cas3 HD-nuclease and SF2 helicase domains, and how the >500 kDa bulk of Cas3-Cascade complex translocates ssDNA. All of these bacteria use a Cascade of five protein subunits, Cas5, 6, 7, 8e (Cse1) and Cse2, previously called CasA-E. DNA nicking by the Cas3 HD-nuclease is directed to the non-targeted DNA strand (NTS) in the R-loop, which forms a displaced ssDNA ‘loop’. Exactly how this is achieved is not clear. Association of Cas3 with the Cse1 subunit of Cascade places the Cas3 HD site distal to the NTS, although a bulge in NTS DNA caused by Cse1 binding might account for this, loading Cas3 on ssDNA HD-first. In this scenario the initial HD-nuclease nicking of ssDNA by Cas3 bypasses the SF2 helicase domains. In cryo-EM analyses [26,35] this is sufficient to re-locate ssDNA to a position much closer to the RecA domains that power the ATP-dependent translocation of Cas3 along ssDNA. An arginine rich channel (ARC), comprising arginine residues that are highly conserved in Cas3 proteins across species, apparent in Cas3 structures (Figure 2) may aid stable ‘capture’ of the ssDNA to initiate translocation.

### 2.2. Cas3 Translocase/Helicase in a CRISPR Interference Machine

Cas3 loads onto ssDNA and translocates it using ATP-hydrolysis [36]. In this way it can separate DNA duplex strands as a helicase, and may also be able to displace other DNA binding proteins during translocation, similarly to other translocases [36,37,38,39]. After initial DNA nicking by Cas3 HD-nuclease, Cas3 translocates DNA with 3’ to 5’ directionality generating ssDNA gaps from a Cascade-R-loop formed on duplex DNA, which is detectable in single-molecule experiments using GFP-RPA [27]. Single-molecule studies of Cas3 from *E. coli* and *T. fusca* show translocation over thousands of nucleotides at mean velocities of 89-316 bp/s [27,40]. *E. coli* Cas3 was found to rapidly translocate away from Cascade, while Cascade remained bound to the target site. In this system the interaction of Cas3 with Cascade, and its subsequent translocation away from Cascade, nucleolytically destroys the substrate (ssDNA) onto which Cas3 loads at the target site, such that no re-association of Cas3 and that same Cascade was observed [27,41]. However, when *T. fusca* Cas3-Cascade was used half of the translocating Cas3 molecules remained associated with Cascade [40]. In this system it is proposed that sustained association of Cas3-Cascade remains essentially fixed in place, as DNA moves relative to it by ‘reeling’ or ‘looping’ powered by ATP-dependent ssDNA translocation of Cas3 [28,40]. The dissociation of *T. fusca* Cas3 from Cascade then resulted in Cascade re-binding to DNA target sites. Reeling of NTS DNA through Cas3 resembles mechanisms of DNA translocation by the DNA replication-recombination-repair helicases PcrA and RecBCD (among others), although with significant differences in DNA translocation velocity and step-size.

DNA targeting by Cascade is pivotal for Type 1 CRISPR systems because it triggers the destructive catalysis of Cas3. Initial collision of Cascade complexes with DNA then requires transfer to target sites that are defined by a trinucleotide PAM sequence—5’-AAG in *E. coli*—that will direct Cascade to ‘lock-on’ to DNA if there is complementary crRNA-DNA sequence for R-loop formation. Target site identification follows biophysical principles established for other site-specific DNA targeting enzymes, which are conceptualized or described in several possible ways—the reader is directed to two references in particular, [42,43], for interesting and accessible accounts of these processes. For Cascades, there are currently some areas of disagreement arising from analyses of target site recognition. In TIRF and FRET studies, *E. coli* Cascade favored 3-dimensional (3-D) diffusion to sample physical space for DNA target sites, analogous to it ‘jumping’ at DNA [27,44]. On sensing a PAM this Cascade dwells to establish if R-loop formation is possible by RNA-DNA base-pairing. In this model the PAM allows Cascade to discern kinetically whether the DNA is a *bona fide* target, or not—summarized in Figure 6 of reference [44]. A similar study using *T. fusca* Cascade favored facilitated diffusion of Cascade over 1-dimension (1-D) [40], in which Cascade essentially ‘slides’ or ‘hops’ along DNA while determining target sites. This also highlighted the importance to the process of five conserved lysine/arginine amino acid residues within the Cascade structure, which is contrasted with *E. coli* Cascade in which three of these five residues are present, a possible factor explaining the discrepancy in behaviors of these two Cascades. Another factor could be that analysis of the 1-D target site search by *T. fusca* used DNA that lacked an identifiable RNA-DNA sequence complementarity that would allow R-loop formation, despite PAMs being present. In any event, successful engagement of Cascade with DNA target recruits the Cas3 translocase-nuclease.

### 2.3. Cas3 Translocase/Nuclease in a CRISPR Interference-Adaptation Machine

DNA reeling by PcrA displaces RecA protein from DNA during DNA replication-repair [45], giving precedence to a possible role for Cas3 reeling in removing bound proteins during CRISPR interference reactions. This aspect of Cas3 is particularly interesting—biophysical data indicates that *T. fusca* Cas3 and Cas3-Cascade are ineffective at removing ectopic protein ‘roadblocks’ from DNA, including RNA polymerase barriers [40] that are most relevant to bacterial chromosome dynamics [46]. However, addition of the CRISPR adaptation complex Cas1-Cas2 to Cas3-Cascade, forming a new machine called the Primed Adaptation Complex (PAC) triggered displacement of RNA polymerase 63% of the time [40]. In this system it is not known if association of Cas1-Cas2 activates the motive power of Cas3 as a DNA translocase, or if Cas1-Cas2 itself modifies some aspect of the PAC or the roadblock to facilitate its displacement. In *Pseudomonas* the nuclease activity of the fused Cas2-Cas3 protein is inhibited strongly by association with Cas1, but this effect is counteracted when the Cas1-Cas2/3 complex encounters Cascade (called ‘Csy’ in this system) that is bound to a target DNA [47].

Physical and functional interaction of the *T. fusca* PAC and Cas1-Cas2/Cas3/Cascade more generally in other systems provides the important biological role linking interference with DNA fragment capture and integration reactions of ‘adaptation’—recently reviewed [48,49,50]. Physical and functional coupling of interference with adaptation in a PAC can re-cycle DNA from invader into CRISPR loci, updating immunity in ‘primed’ or ‘targeted’ adaptation [51,52,53,54,55,56,57,58,59]. FRET measurements indicated that Cas3 DNA reeling is stimulated by Cascade but that Cas3 nuclease activity is weak, when compared to nuclease activity recorded from bulk/ensemble biochemical assays [28]. However, in this study there were no measurements made of the PAC, by addition of Cas1-Cas2. It would be useful to determine if nuclease activity within the PAC is stimulated relative to Cas3-Cascade and, if so, whether this is attributable to Cas3, Cas1-Cas2, or both—there is data suggesting that Cas1-Cas2, and Cas2 alone, have nuclease activity [60,61,62]. This would help to understand further the molecular events that underpin the various stages of primed adaptation.

## 3. Characteristics of Cas3-Cascade Used for Genetic Editing 

The critical importance of Cas3-catalyzed DNA destruction for the functioning of natural CRISPR systems [9,21] is also highlighted in its manipulation for genetic editing reactions. By deleting Cas3 from *E. coli* cells (Δ*cas3*) it has been possible to steer Cascade alone to DNA targets for non-destructive gene repression, targeting ectopic reporter genes (e.g., GFP) or native genes [63]. Bacterial Cas3 has been used alongside Cascade in various human cell types, giving ‘proof-of-principle’ that Cas3-Cascade is functional for targeted genetic insertions or deletions (‘indels’) when delivered into cells as proteins with nuclear localization signals, or expressed endogenously [64,65,66]. One encouraging trait arising from Cascade-Cas3 editing reactions, if compared with Cas9, is the low rate of off-target effects observed in a study using *T. fusca* Cascade-Cas3 [64], attributed to the stringency by which Cascade interrogates DNA for a target, and the requirement for it to ‘lock-on’ to the DNA target before Cas3 can be recruited to start its nuclease rampage. In human embryonic stem cells it was reported that the editing efficiency—reported by disruption of green fluorescent protein (GFP) maximally at 13%—was determined by available Cascade molecules and was unaffected by increasing availability of Cas3 [64]. These reports indicate the emergence of Cascade as a valuable method for targeted genome-editing, offering advantages over simpler Cas9/Cas12 editing enzymes that override the disadvantage of Cascade’s multi-subunit structure. The diversity of Cascades available from natural sources offers many opportunities for fine-tuning Cascade for this purpose. For example, a study using different Cascades fused to FokI restriction endonuclease identified that DNA targeting and editing efficiency was several-fold more effective if the Cascade used in human U20S, HEK293 or HeLa cells was from a *Pseudomonas* species rather than from other bacteria [66].

In each of these genome-editing studies Cas3 has generated large regions of genetic deletion, meeting expectations arising from knowledge of its mechanism and catalytic power. Deletions of up to 200 kb were reported from *T. fusca* Cas3-Cascade when expressed from genes in human HEK293 cells [66], and similar sized deletions were observed from *E. coli* Cas3-Cascade, also expressed from transfected DNA constructs [65]. In the latter study Cascade-Cas3 was successful at achieving an ‘exon skipping’ reaction targeted to human dystrophin encoding gene, reactions thought to have potential as a therapeutic treatment for muscular dystrophies [67]. The ability of Cascade-Cas3 to catalyze high stringency-high throughput DNA deletion within human cells is a useful new tool to understand large chunks of the genome.

## 4. Interaction of Cas3 with HtpG and Other Host Proteins That Are Not Part of CRISPR Systems

A genome-wide protein interaction study using >4000 immobilized *E. coli* proteins identified seven proteins that physically interacted with hexahistidine tagged Cas3 (at that time called YgcB) [68]; GroL, DnaE, GyrA, AceE, IbpA, CrfC, and MdoD each co-eluted with Cas3 immobilized on Ni-NTA beads. More generally across bacteria and archaea, functional interactions of CRISPR systems with DNA repair systems are important for efficient functioning of CRISPR immunity, especially for adaptation reactions that build CRISPR loci from captured invader or host DNA fragments [61,69,70,71,72,73].

**Figure 3:**
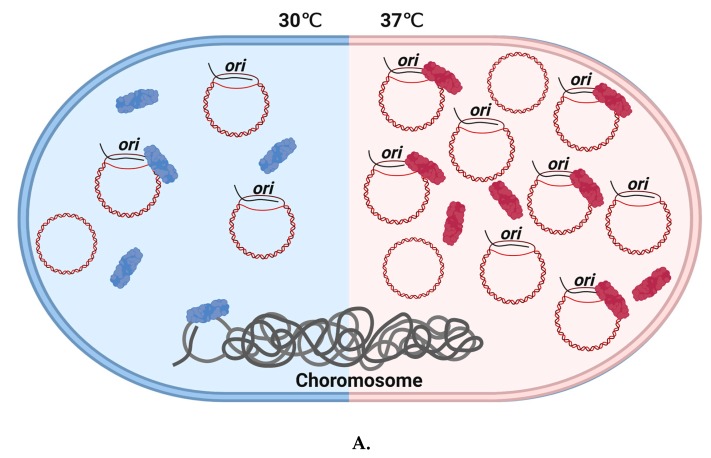

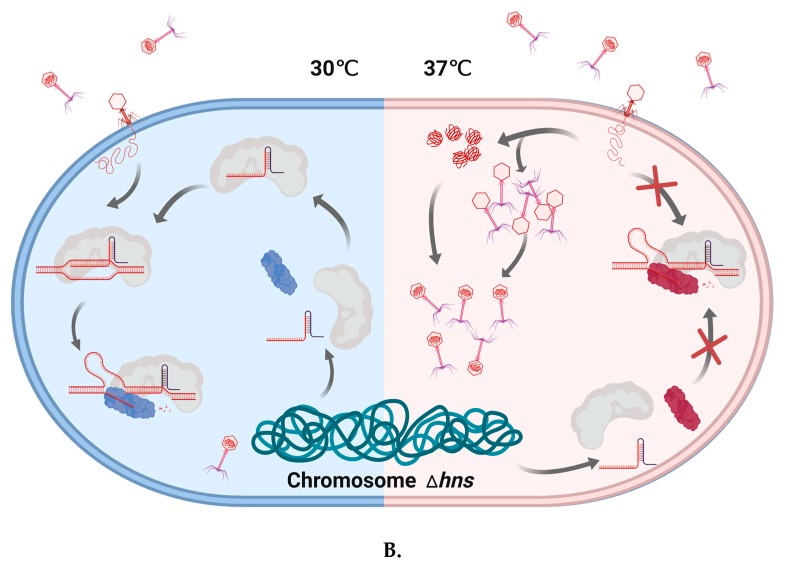
Temperature-dependent Cas3/CRISPR functions in *E. coli* and the *E. coli* Δ*hns* strain. **A.** Cas3 stimulates uncontrolled ColE1 plasmid replication in a temperature-dependent manner. ColE1 plasmid yield was stimulated by Cas3 in cells at 37 °C but not at 30 °C. This requires a functional helicase domain. We speculate that this phenomenon may indicate a possible change in Cas3 conformational ‘state’, illustrated by coloring Cas3 in blue ‘demotivated’ or red ‘motivated’. At 37 °C, ‘motivated’ Cas3 may interact with R-loop formation in *ori* by dissociating RNA II from the complementary strand, and lead to increased plasmid replication in vitro. **B.** Temperature impacts CRISPR function in *E. coli* cells lacking H-NS. This Δ*hns* strain at 30 °C can defend against invader DNA during phage infection, even though Cas3 is in ‘unmotivated’ state. However, Δ*hns* strain cannot survive under infection pressure at 37 °C.

Stable functioning of Cas3 as an essential part of CRISPR immunity processes requires co-expression of a chaperone protein HtpG in *E. coli* and probably in other bacteria [74], although HtpG is not widely distributed across archaea. Functional interaction between Cas3 and HtpG is observed in vivo; CRISPR immunity against lysogenization by λ prophage at 32 °C was lost when HtpG was lacking (Δ*htpG*), an effect suppressed by Cas3 over-expression. The efficacy of the CRISPR immunity against λ phage infection is also temperature-dependent—cells lacking HNS protein (Δ*hns*) that are engineered to target λ phage are effective at resisting infection at 30 °C but not 37 °C (Figure 3), but cells lacking HtpG and HNS are phage-sensitive. Over-expression of Cas3 or HtpG rescued this phage sensitivity, but only at 30 °C—the same cells remained sensitive at 37 °C [75]. The reason for Cas3 instability in Δ*hns* cells at 37 °C is not known, but may be useful in revealing other roles for Cas3 in cells, and it will help to have a better understanding of the molecular basis for interaction between Cas3 and HtpG.

## 5. UnPACking of Cas3 from Cascade—Activities with RNA 

An interesting set of observations has been made implicating Cas3 in several other cellular events that center on, or are at least associated with, regulatory processing of RNA molecules. In one example, ectopic over-expression of Cas3 in *E. coli* cells stimulated uncontrolled DNA replication of plasmids from their ColE1 replicons, but had no such effect on non-ColE1 plasmids [76]. These plasmids use R-loop at *ori* to initiate replication formed by plasmid encoded RNA molecules, one that primes replication (RNAII) and the other that prevents priming (RNAI) [77]. Cas3 stimulates priming of replication, dependent on its ATPase/translocase activity independently of Cascade [76]. It is proposed that Cas3 can specifically target RNAI-RNAII molecules for dissociation, liberating RNAII to pair with DNA at *ori* and priming replication. This unusual property of Cas3 to stimulate uncontrolled plasmid replication has been shown to be temperature-dependent, increased at 37 °C and decreased at 30 °C (Figure 3). Reduced or inhibited ATPase/helicase activity of Cas3 at 30 °C was found responsible for this effect, and Δ*hns* mutation was found to indirectly decrease plasmid copy number at 30 °C [78].

Purified Cas3 from *E. coli* and the archaeal species *Methanothermobacter thermautotrophicus* can manipulate RNA molecules in vitro. In common with helicase/translocase enzymes of the RecQ family [79,80,81], Cas3 can anneal nucleic acid strands as well as unwind them—purified Cas3 is especially robust at annealing RNA strands into plasmid DNA (i.e., R-loop formation) [82]. This activity required the Cas3 HD domain but is independent of ATPase activity—HD domain-catalyzed nicking of DNA is thought to facilitate RNA-DNA annealing by relaxing plasmid supercoiling. It is difficult to interpret what specific role this in vitro activity of Cas3 may have in cells, if any, but it does support ideas that Cas3 may transmit RNA signals into cellular networks more widely than CRISPR systems. Analysis of the organization of the *E. coli* MG1655 CRISPR-Cas system identified a promoter, ‘anti-Pcas’, located just downstream of the *cas3* gene coding sequence (see Figure 1B), and which generates detectable RNA transcript of 150–200 nucleotides [83] that would overlap with the *cas3* transcript. The authors of that study predict that the anti-Pcas transcript may form an elaborate folded structure, which is reminiscent of riboswitches and other regulatory RNAs. The significance of this, and any role for Cas3 in its putative function, is not known.

Regulatory roles for CRISPR systems are identified in several contexts [84,85,86]. The DNA targeting capabilities of Cascade, and observations that CRISPR systems frequently capture ‘self’ DNA from host chromosomes as well as ‘non-self’ invader DNA, indicate that CRISPR systems may regulate gene function as transcription factors [87,88]. Cascade gene targeting has been used for ectopic control of gene expression [89,90]. Cas3 influences group behavior of *Pseudomonas* cells—formation of biofilms is inhibited in response to lysogeny by phage DMS3, a response that requires Cas3 to be fully functioning for HD-nuclease and helicase activities [91]. In these cells chromosomal sequences partially match sequences of DMS3 that might allow binding of Cascade that is sufficient to trigger the DNA nicking step of Cas3 and some ssDNA formation at the Cascade target site. In this model the ssDNA generated may be enough to trigger the loading of the DNA recombinase RecA, triggering a DNA-repair “SOS” response that is known to be able to block biofilm formation [92].

As would be expected for the ‘arms-race’ between viruses and their hosts, the crucial roles for Cas3 in CRISPR immunity and suppressing biofilm formation have driven evolution of inhibitors, which in *Pseudomonas* virus JBD5 is AcrF3 protein [93,94]. The molecular details of interaction between AcrF3 and Cas3 are fascinating, explaining how the inhibitor switches off Cas3 function, informed by the detailed analyses of Cas3 structure-function, in isolation and in association with Cascade and in the PAC. There is likely to be plenty more to learn about Cas3 function as a nuclease, translocase, and possibly as an RNA processing enzyme.
